# Perceval Implantation and Ascending Replacement: Which Should Be Performed First?

**DOI:** 10.1055/s-0041-1730008

**Published:** 2021-10-07

**Authors:** Giuseppe Santarpino, Luigi Specchia, Pasquale Mastroroberto, Renato Gregorini

**Affiliations:** 1 Department of Cardiac Surgery, Anthea Hospital, Gruppo Villa Maria Care & Research, Bari, Italy; 2 Department of Cardiac Surgery, Paracelsus Medical University, Nuremberg, Germany; 3 Department of Experimental and Clinical Medicine, Cardiac Surgery Unit, University “Magna Graecia” of Catanzaro, Catanzaro, Italy; 4 Department of Cardiac Surgery, Città di Lecce Hospital, Gruppo Villa Maria Care & Research, Lecce, Italy

**Keywords:** aortic aneurysm, aortic valve and root, heart valve surgery

## Abstract

The use of sutureless prostheses has expanded due to their ability to reduce surgical times, thus favoring their implantation in high-risk patients. It is not uncommon that these patients have an ascending aortic aneurysm requiring treatment with a vascular prosthesis; therefore, using a sutureless aortic valve may be associated. To date, however, little is known about the time sequence of this intervention, that is, if sutureless implantation should precede or follow that of the vascular prosthesis.

## Introduction

Advances in surgical techniques have made it possible to perform surgical procedures in an increasing number of older and high-risk patients. In order to reduce the time and risks of surgery, accurate preoperative assessment and planning are mandatory. In patients with aortic valve disease and ascending aortic aneurysm, the use of sutureless prostheses can facilitate the procedure by minimizing surgical times, particularly the implantation phase. We report the case of a patient undergoing sutureless aortic valve implantation in combination with ascending aorta replacement, providing a detailed description of the surgical technique and explaining which of the two prostheses (vascular or valvular) to implant first.

## Case Presentation

A 71-year-old patient with history of aortic valve surgery was referred to our Center. He underwent aortic valve replacement for severe aortic stenosis at 53 years with a Toronto SPV (St Jude Medical, Saint Paul, Minnesota) bioprosthesis (27 mm). Ten years later, the patient was reoperated due to prosthetic valve dysfunction and received a Mitroflow (LivaNova PLC, London United Kingdom) bioprosthesis (21 mm). At the time of this second intervention, the maximum diameter of ascending aorta was approximately 45 mm, and it was decided not to replace the ascending aorta in combination with the valve implant procedure. In 2019, after regular follow-up visits, degeneration of the bioprosthetic valve was observed with severe aortic valve insufficiency and an ascending aorta with maximum diameter reaching 51 mm. Plans were made for a third aortic valve replacement combined with replacement of the ascending aorta. The choice between surgery and transcatheter aortic valve implantation was discussed within the heart team. Despite a high-operative risk (the European System for Cardiac Operative Risk Evaluation [EuroSCORE] II: 11.33%: 71-year-old, male sex, previous cardiac surgery of New York Heart Association [NYHA] class II, left ventricular ejection fraction 31–50%, moderate pulmonary hypertension, and surgical procedure on the thoracic aorta), the patient was scheduled for surgery due to ascending aortic aneurysm. Complete resterntomy was planned with the possibility of central arterial and venous cannulation due to bad adhesions between the heart and the sternum observed at preoperative computed tomography. Positioning of a Perceval sutureless prosthesis (LivaNova PLC, London, United Kingdom) was also planned to reduce the procedural time.

Based on available data from the literature, 1 it was deemed opportune to first implant the vascular prosthesis with construction of the proximal aortic anastomosis, followed by implantation of the aortic valve prosthesis.

**Fig. 1:**
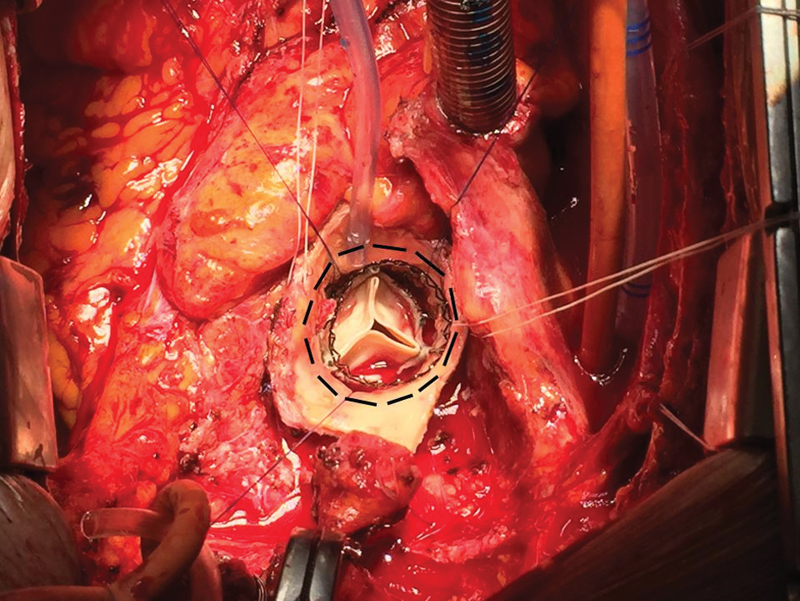
Perceval aortic valve implanted with visible free margin between the stent and the edge of the aorta to be sutured (black dashed line).

**Fig. 2:**
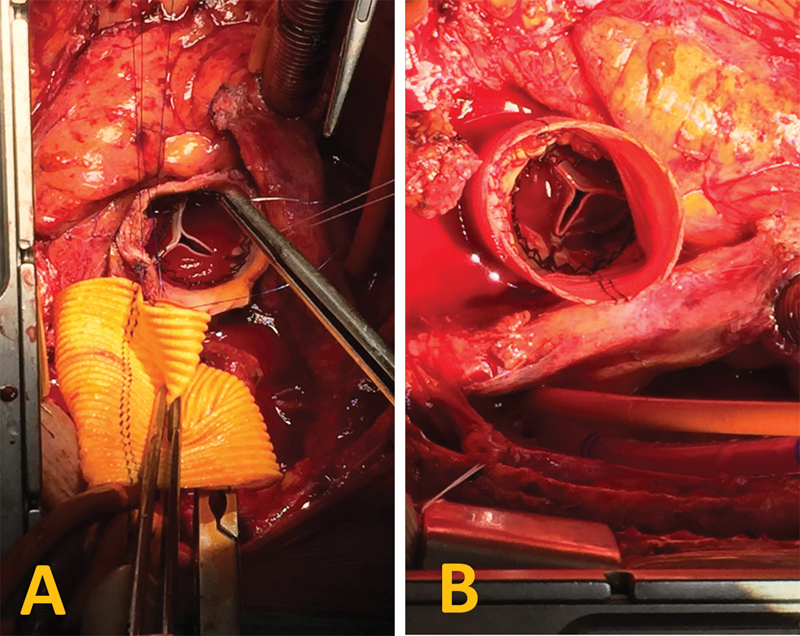
( **A** ) The vascular prosthesis prior to anastomosis after Perceval aortic valve implantation. ( **B** ) The Perceval aortic valve within the vascular prosthesis after anastomosis was completed.

After resternotomy, arterial cannulation of the aortic arch was performed, and venous cannulation via the right atrium was also performed with aortic cross-clamping and infusion of direct warm blood cardioplegia into the coronary ostia due to severe aortic valve regurgitation. After excision of the ascending aortic aneurysm down to the sinotubular junction and explantation of the degenerated valve prosthesis, it was noticed that there could be enough space between the upper edge of the stent of the sutureless valve in the aortic root and the margin of the aorta to perform the proximal anastomosis of the vascular prosthesis. Before implantation of the valve prosthesis, this was only hypothesized but considering the possibility of easily explanting the prosthesis, if our theory turned out to be incorrect, 2 after appropriate sizing, we decided to implant a Perceval prosthesis of size S using standard technique. 3 Our hypothesis was then confirmed as there was enough margin between the stent and the aortic cuff for anastomosing the vascular prosthesis ( Figs. 1 and 2 ). Vascular anastomoses were performed proximally and distally according to our practice with Prolene 5/0, and needle RB-1 Plus (Ethicon, Johnson & Johnson, New Brunswick, NJ). The vascular prosthesis was of the Hemashield Platinum type (Intervascular, La Ciotat Cedex, France) with a diameter of 30 mm.

The whole operation lasted for 270 minutes, with cardiopulmonary bypass and aortic cross-clamping times of 137 and 124 minutes, respectively. The postoperative period was uneventful. The patient signed a consent for the scientific use of the clinical data.

## Discussion

Advances in surgical techniques along with the introduction into the market of sutureless aortic prostheses have made it possible to perform surgical procedures in an increasing number of older and high-risk patients with aortic valve disease. The use of sutureless aortic valves has been shown to simplify interventions, thus reducing procedural times by up to 40% and lowering surgical risk. 4 For patients who cannot undergo a transcatheter procedure due to the presence of an ascending aortic aneurysm and classified as being at high risk by the heart team, the combination of a vascular prosthesis with a sutureless aortic valve can be a valuable compromise for making procedural times shorter.

Our case shows that, by displaying sufficient space in the aortic root between the Perceval valve stent housing and the edge of the ascending aorta, valve prosthesis implantation can be performed prior to vascular prosthesis implant.

In contrast, if there is not enough space, the risk of passing the suture through the stent mesh with subsequent stent distortion is very high. In such circumstances, we believe that the best strategy is to perform first implantation of the vascular prosthesis followed by implantation of the sutureless prosthesis into the anastomosed vascular prosthesis. 1

Therefore, a question arises regarding which should be performed first, Perceval valve implantation or ascending aorta replacement? We believe the answer is that both are feasible (i.e. the valve prosthesis can be implanted either before or after the vascular prosthesis). However, the crucial factor when choosing which prosthesis to implant first is having enough cuff of aorta far from the stent, so as to avoid interference between the suture and the stent mesh.
